# American Help for Child Welfare

**Published:** 1920-10-09

**Authors:** 


### American Help for Child Welfare.

Swansea was one of the towns which participated recently in the generous aid given by the American Red Cross Society to maternal and infant-welfare work in this country. A grant of £2,000 was made towards the establishment of a new maternity home to accommodate nine lying-in cases, at Gore Terrace, Swansea. The new home has been inspected by Miss J. Halford, the Secretary of the National League for Maternity and Child Welfare, who has since written to Dr. Evans, the Borough Medical Officer of Health, expressing delight at all she saw there, and promising, on behalf of the American Red Cross, a further grant of £500.

